# Comparative analysis of 2 approaches to monitor countries’ progress towards full and equal access to sexual and reproductive health care, information, and education in 75 countries: An observational validation study

**DOI:** 10.1371/journal.pmed.1004476

**Published:** 2024-12-31

**Authors:** Jewel Gausman, Richard Adanu, Delia A. B. Bandoh, Neena R Kapoor, Ernest Kenu, Ana Langer, Magdalene A. Odikro, Thomas Pullum, R. Rima Jolivet

**Affiliations:** 1 Guttmacher Institute, New York, New York, United States of America; 2 Maternal and Child Health Nursing Department, School of Nursing, University of Jordan, Amman, Jordan; 3 Department of Global Health and Population, Harvard University T.H. Chan School of Public Health, Boston, Massachusetts, United States of America; 4 Department of Population, Family, and Reproductive Health, University of Ghana School of Public Health, Accra, Ghana; 5 Department of Epidemiology and Disease Control, University of Ghana School of Public Health, Accra, Ghana; 6 ICF International/Demographic and Health Surveys Program, Rockville, Maryland, United States of America

## Abstract

**Background:**

Sustainable Development Goal (SDG) Indicator 5.6.2 is the “Number of countries with laws and regulations that guarantee full and equal access to women and men aged 15 years and older to sexual and reproductive health care, information, and education.” This indicator plays a key role in tracking global progress toward achieving gender equity and empowerment, ensuring its validity is essential. Significant challenges related to the indicator’s calculation have been noted, which have important implications for the indicator’s validity in measuring progress towards meeting the SDG target. Recommendations have been made to revise the scoring of the indicator. This study examines the indicator’s validity by proposing a revision to the indicator’s calculation that addresses these global concerns and comparing the resulting values.

**Methods and findings:**

This is an observational, validation study which used secondary data from the 2022 United Nations Population Fund’s Sexual and Reproductive Health and Rights Country Profiles from 75 countries. To address global recommendations, we proposed making 2 changes to the indicator’s calculation. First, we re-expressed all barriers and enablers to take positive values. Second, we used a weighted additive approach to calculate the total score, rather than the mean of the 13 individual component scores, which assigns equal weight to the substantive domains rather than the components. Our main outcome measures are the indicator values obtained from both scoring approaches examined. We assessed the indicator’s convergent validity by comparing the value obtained using the indicator’s current formula to the proposed formula using the Bland–Altman approach. We examined and interpreted changes in the indicator’s overall score that result from comparing the existing indicator with the proposed alternative. Differences in the total value of the indicator comparing the alternative versus the current formulation range from −7.18 percentage points in Mali to 26.21 percentage points in South Sudan. The majority of countries (*n* = 47) had an increase in total indicator score as a result of the alternative formula, while 27 countries had a decrease in score. Only 1 country, Sweden, saw no change in score, as it scored 100% of the possible indicator value under both rubrics. The mean difference between the scores produced by the 2 measures is 2.28 suggesting that the 2 methods may produce systematically different results. Under the alternative formulation, the most substantial changes were observed in the scores for “Component 3: Abortion.” The indicator’s current calculation results in 16 countries being assigned a score of zero, for “Component 3: Abortion” which masks important differences in the number of legal barriers present and whether women can be criminally charged for illegal abortion. After re-expressing barriers on a positive scale following the proposed formulation, only 4 countries have a score of zero for Component 3. The main limitation of our methodology is that there is no gold standard for measurement of the phenomenon under study, and thus we are unable to specify with total certainty which indicator performs better.

**Conclusions:**

Our results illustrate underlying challenges with the current indicator formulation that impact its interpretability. The proposed changes could alter the way the current legal landscape governing sexual and reproductive health is understood, thereby pointing to different programmatic and policy priorities that may better support countries in achieving full and equal access to sexual and reproductive health and rights globally.

## Abstract 2

Jewel Gausman and co-workers explore alternative approaches to measure progress towards equal acess to sexual and reproductive healthcare.

## Author summary

**Why was this study done?:**

- Sustainable Development Goal (SDG) Indicator 5.6.2 is defined as the “number of countries with laws and regulations that guarantee full and equal access to women and men aged 15 years and older to sexual and reproductive health care, information and education.”
- With its key role in measuring progress towards achieving gender equity and empowerment for all, ensuring the validity of this indicator is essential.
- Past research has been critical of the construction of the indicator, but no previous studies have attempted to validate its calculation.

**What did the researchers do and find?:**

- We addressed the challenges with the calculation of the indicator that had been previously identified in the published literature and proposed an alternative method of calculation.
- We calculated the value of SDG Indicator 5.6.2 and iteratively compared it to the proposed alternative using data from 75 countries globally.
- Our results show that the 2 different measurement approaches for the same underlying construct produce systematically different values, supporting a need to revisit the formulation of the existing indicator.

**What do these findings mean?:**

- Our proposed revisions may improve the overall stability and interpretability of the indicator’s value across countries.
- The changes proposed to the indicator’s calculation suggest different priorities for action to drive progress in achieving full and equal access to sexual and reproductive health care, information, and education.
- Our study is limited by the fact that there is no gold standard in measurement of the policy and legal environment, thus all measures considered are proxies for the underlying construct.

## Introduction

Sustainable Development Goal (SDG) Indicator 5.6.2 is the “number of countries with laws and regulations that guarantee full and equal access to women and men aged 15 years and older to sexual and reproductive health care, information and education,” which represents the first global attempt to operationalize measurement of the priorities set in the Programme of Action of the International Conference on Population and Development (ICPD) and the Beijing Platform for Action [1]. This indicator was prioritized as a core measure to monitor progress towards achieving the objectives outlined in “Strategies toward Ending Preventable Maternal Mortality” (EPMM), the strategic framework for maternal health in the SDG period [2], and chosen for its utility to track progress toward empowering women and girls, families, and communities, a key theme of the EPMM report [3]. Existing literature has been critical of the indicator’s formulation, raising important questions about its validity [4]. With its key role in measuring progress towards achieving gender equity and empowerment for all, ensuring the validity of this indicator is essential [5].

Laws and regulations enumerated in the indicator fall into the following 4 categories: maternity care services, contraception and family planning, comprehensive sexuality education and information, and sexual health and wellbeing. These thematic domains (referred to as “sections” in the indicator’s metadata) are represented by a total of 13 individual components that were selected to reflect both a broad spectrum of topics that are critical from a substantive perspective as well as subjects commonly included in national legal and regulatory frameworks [6]. Each component comprises a set of enabling laws, which represent positive laws that are codified in a country’s national legal framework, and a set of legal barriers, which are thought to undermine the positive impact that the enabling laws may have on ensuring full and equal access to sexual and reproductive health care, information, and education [7]. The summative score of the indicator is interpreted by UNFPA as a country having achieved a certain “percent of enabling laws and regulations for full and equal access for women and men aged 15 years and older to sexual and reproductive care, education, and information.” [8]

Content validity is predicated on how well and completely the items in the measure reflect the scope of constructs represented within the phenomenon to be measured [9]. Those involved in the process of creating the original indicator described pressure to reduce the number of thematic domains that were included from a longer list that reflected the full breadth of ICPD topics [10]. The 13 components included in the final operationalization of Indicator 5.6.2 were intended to reflect key parameters from international consensus documents and human rights standards and were designed to be representative, but not exhaustive [7], and therefore the measure may not reflect the full content domain. The indicator has been revised through multiple expert consultations to simplify the survey and scoring [1]. In particular, survey questions were reduced to yes/no responses. While serving to facilitate data collection and analysis, doing so may entail significant loss of meaning in measuring a complex policy environment [10].

Construct validity reflects the accuracy with which an indicator captures the phenomena it intends to measure. There have been calls in the literature to revise how SDG 5.6.2 is calculated [4]. In the previous version of the metadata, the formula for subtracting barriers from enablers “Component 3: Abortion” caused its value to be a negative number in some countries, and thereby uninterpretable on the indicator’s range of possible values defined between zero and 100%. Past critiques have led to recent revisions in the formula for calculating “Component 3: Abortion” to ensure the value cannot be negative.

While the possibility of the indicator achieving a negative value has been addressed, there are other important critiques related to subtracting barriers from enablers in the indicator’s calculation. First, concerns have been raised about the indicator’s method of calculation in that it subtracts barriers from enablers in a way that is sensitive to the number of barriers and enablers included. In future revisions of the indicator, both the number of enablers and barriers could theoretically expand, which is an especially important consideration given the critiques related to the indicator’s content validity. Further, any barrier can be re-expressed as the absence of an enabler, making the distinction between enablers and barriers arbitrary. For example, the presence of a plural legal system is specified as a barrier in many of the indicator’s components. Plural legal systems are legal systems in which multiple sources of law coexist, often due to colonial inheritance, religion, and other sociocultural factors [11]. The formula for the current indicator subtracts one point if a plural legal system is present.

Another concern is that the total score reflects the mean obtained from the 13 individual components, not the 4 substantive domains. This approach inadvertently assigns more importance to domains with more components than others, rather than giving all domains equal weight.

We argue that the indicator’s current method of calculation compromises its construct validity. To our knowledge, this is the first paper to assess the validity of SDG 5.6.2 using country-level data. The aims of this paper are (1) to demonstrate the challenges that arise due to the indicator’s current method of calculation; (2) to compare the existing approach to a proposed simplified method that addresses the challenges identified; and (3) to examine the implications of the 2 different scoring approaches on the indicator’s interpretation.

## Methods

### Data

This study used secondary data obtained from UNFPA’s Sexual and Reproductive Health and Rights Country Profiles [8]. These data are collected from responses to a national survey of official government and bilateral stakeholders in each country [3].

All 75 countries that reported data were included in the study. The Institutional Review Board (IRB) of the Harvard T.H. Chan School of Public Health approved this study on 4 September 2019.

### Analysis

We first calculated the value of SDG Indicator 5.6.2 using the formula provided in the indicator’s metadata (the current formula) [7]. The 4 thematic domains (sections), 13 components, and their associated enablers and barriers that comprise the indicator’s value are provided in Table 1.

**Table 1:** SDG Indicator 5.6.2 metadata: Extent to which countries have laws and regulations that guarantee full and equal access to sexual and reproductive health care, information, and education for women and men aged ≥15 years.

Section	Enablers	Barriers
**Section 1: Maternity Care**		
Component 1: Maternity Care	Enabling law present	Plural legal system contradicts enabling law Restrictions based on age, marital status, or third party authorization
Component 2: Life Saving Commodities	13 commodities identified	None identified
Component 3: Abortion	Abortion is permitted: To save a woman’s life To preserve a woman’s physical health In case of fetal impairment In case of rape	Abortion is legal on some or all legal grounds, but (1) authorization of medical professional is required, (2) judicial consent is required for minors, or (3) husband’s consent is required A provider or person who helps a woman obtain an abortion can be charged for illegal abortion
Component 4: Post-Abortion Care	Enabling law present	Plural legal system contradicts enabling law Restrictions based on age, marital status, or third party authorization
**Section 2: Contraceptives and Family Planning**		
Component 5: Contraceptive Services	Enabling law present	Plural legal system contradicts enabling law Restrictions based on age, sex, marital status, or third party authorization
Component 6: Contraceptive Consent	Enabling law present	• Plural legal system contradicts enabling law
Component 7: Emergency Contraception	Enabling law present	Plural legal system contradicts enabling law Restrictions based on age, marital status, or third party authorization
**Section 3: Sexuality Education**		
Component 8: Sexuality Education Curriculum Laws	Enabling law present	• Plural legal system contradicts enabling law
Component 9: Sexuality Education Curriculum Topics	8 topics identified	None identified
**Section 4: HIV and HPV**		
Component 10: HIV Counseling and Test Services	Enabling law present	Plural legal system contradicts enabling law Restrictions based on age, sex, marital status, or third party authorization
Component 11: HIV Treatment and Care Services	Enabling law present	Plural legal system contradicts enabling law Restrictions based on age, sex, marital status, or third party authorization
Component 12: HIV Confidentiality	Enabling law present	Plural legal system contradicts enabling law Restrictions based on age, sex, marital status, or third party authorization
Component 13: HPV Vaccine	Enabling law present	• Plural legal system contradicts enabling law

From the indicator’s metadata (5):

For each of the 13 components, information is collected on the existence of (i) specific legal enablers (positive laws, and regulations) and (ii) specific legal barriers. Such barriers encompass restrictions to positive laws and regulations (e.g., by age, sex, marital status, and requirement for third party authorization), as well as plural legal systems that contradict coexisting positive laws and regulations. For each component, the specific enablers and barriers on which data are collected are defined as the principle enablers and barriers for that component. Even where positive laws are in place, legal barriers can undermine full and equal access to sexual and reproductive health care, information, and education; the methodology is designed to capture this.

The percentage value reflects a country’s status and progress in the existence of national laws and regulations that guarantee full and equal access to sexual and reproductive health care, information, and education. By reflecting the extent to which countries guarantee full and equal access to sexual and reproductive health care, information, and education; this indicator allows cross-country comparison and within-country progress over time to be captured.

Plural legal systems are defined as legal systems in which multiple sources of law coexist. Such legal systems have typically developed over a period because of colonial inheritance, religion, and other sociocultural factors.

The indicator’s current formula subtracts the proportion of potential barriers that are documented from the proportion of potential enablers that are documented in that country for each of the 13 components defined in the indicator’s metadata. The result is then multiplied by 100 to produce a percent. Each component is calculated individually and weighted equally, and the indicator value is calculated as the arithmetic mean of the 13 components [7].

The indicator metadata refers to 4 “sections,” which correspond to the 4 substantive domains. The indicator’s metadata specifies that section mean can be calculated as the mean of its constituent components, and have value on their own, but the mean section scores are not included in the overall value of the indicator. While all of the components generally follow the same formula, the formula for calculating the score for C3: Abortion is different from the others given the desire to avoid a negative score, as the number of barriers identified outweigh the enablers. This formula has recently been revised from a previous version in which the value for C3: Abortion was able to take on a negative value under some circumstances. Equations for calculating each component score and the total indicator score following the current formulation can be found in Box 1. The full calculation method for the current scoring of the indicator is provided in “**Documentation for current SDG Indicator 5.6.2 scoring and alternative calculation in** S1 Text.”

**Box 1: Current Formula for Calculating SDG Indicator 5.6.2**

*Formula for All Components except for C3*: *Abortion*

$$Ci=\left(\frac{ei}{Ei}-\frac{bi}{Bi}\right)\;\times 100$$

*Formula for “Component 3*: *Abortion*”

$$Ci=\frac{ei}{Ei}\left(1-\frac{bi}{Bi}\right)\;\times 100$$

*Formula for Total Indicator Score*

$$Score=(\sum_{i=1}^{13}C_{i})/13$$

*C*_*i*_ is the score for component *i*; *E*_*i*_ is total number of enablers identified in component *i*; and *e*_*i*_ is number of those enablers that exist in a given country. *B*_*i*_ is total number of barriers identified component *i*, and *b*_*i*_ is number of those barriers that exist in a given country.

Our proposed alternative formula for calculating SDG Indicator 5.6.2 contains 2 major changes. First, to calculate individual component scores, we re-expressed all barriers so that a country is given a score of plus one (+1) if no barrier exists, rather than giving a country a score of negative one (−1) if a barrier is present—as is done following the indicator’s current calculation. In other words, we inversely code the current metadata’s scoring of barriers so that the lack of a barrier receives a positive score, rather than the presence of a barrier receiving a negative score. We believe that this change results in more consistent weighting of barriers and enablers in calculating a component’s score. As in the indicator’s current scoring, “Component 3: Abortion” requires a different formula given the differences in this component’s content because whether criminal charges can apply in the case of an illegal abortion is not contingent upon an enabling law being in place (Barrier #4). Stated differently, in countries where abortion is illegal on all grounds, some countries may criminally charge women for obtaining an illegal abortion, whereas other countries may not. Further, we treat barrier #4 differently because that barrier has a substantive difference from the other barriers, thus limiting the way we can redefine it. The main difference between C3 and the way that we handle the other barriers is that for the other components, the barriers do not apply if there are no enablers present, and the score is zero. The difference with C3 is that only barriers 1–3 are contingent on enablers being present. To accommodate this possibility, and to be true to the substance in the metadata, we believe that there is an important rationale to treat barrier #4 differently.

Second, our alternative formula uses a weighted additive approach that assigns equal weight to each of the 4 sections. Each item in a given component is assigned an equal weight that is inversely proportional to the number of items within the component. The more items in a component, the less weight each individual item contributes to the component score.

Equations for calculating each component score and the total indicator score following the proposed formulation can be found in Box 2.

Our alternate scoring proposal does not alter the substantive content of the indicator so that if the effect of a barrier was contingent upon the presence of an enabler in the indicator’s current formulation, it remains so in our calculation approach. For example, without an enabling law in place there cannot be a plural legal system nor can there be restrictions on age, sex, marital status, or third party authorization.

**Box 2: Alternative Formula for Calculating SDG 5.6.2**

In most settings, the score for Component *i* (i = 1,…,13) is calculated as follows:

$$c_{i}=\frac{e_{i}+B_{i}-b_{i}}{E_{i}+B_{i}}\;\times 100$$

This score is always in a range from 0 to 100, because *e*_*i*_ is in a range from 0 to *E*_*i*_ and *b*_*i*_ is in a range from 0 to *B*_*i*_.

In a context with no enablers, *c*_*i*_ is defined to be to be 0. That is, if *e*_*i*_ = 0, then *c*_*i*_ = 0, regardless of the number of barriers given the inherent dependency in the way that barriers are envisioned in the indicator’s metadata.

An exception is made for C3, related to just the fourth barrier, which is coded as 1 if a woman can be criminally charged for an illegal abortion (and 0 otherwise) in the current formula. The content of the original metadata suggests that this barrier should be considered even within *in a context with no enablers*, as it is not contingent on the presence of an enabler like the other barriers identified. Therefore, we define C3 to be 0, the lowest extreme, only if there are no enablers and the fourth barrier is present. If there are no enablers and the fourth barrier is absent, that is, if there are no enablers present and a woman cannot be criminally charged for an illegal abortion, then the formula becomes

$$c_{3}=\frac{1}{E_{3}+B_{3}}\;\times 100$$

which is the minimum possible non-zero value.

*Formula for Total Indicator Score*

With each component score calculated as above, the alternative calculation for the total indicator score is as below:

$$Total\;Score=\frac{\left(\frac{C1+C2+C3+C4}{4}\right)+\left(\frac{C5+C6+C7}{3}\right)+\left(\frac{C8+C9}{2}\right)+\left(\frac{C10+C11+C12+C13}{4}\right)}{4}$$

*C*_*i*_ is the score for component *i*; *E*_*i*_ is total number of enablers identified in component *i*; and *e*_*i*_ is number of those enablers that exist in a given country. *B*_*i*_ is total number of barriers identified component *i*, and *b*_*i*_ is number of those barriers that exist in a given country.

In our analysis, we explored the indicator’s construct validity by focusing on convergent validity. Convergent validity examines the extent to which the value of one measurement is similar to that of other measures that aim to measure the same underlying construct; this type of validity is commonly assessed when no gold standard measure is available [9]. To do this, we examine differences in the performance of the current and proposed calculations in relation to the specific challenges identified above. We first compared the total indicator scores calculated for SDG Indicator 5.6.2 using the current versus proposed formula across all countries included in the analysis, as well as the differences in scores for each of the 4 sections and 13 components. Last, we examine systematic differences between the measures using a Bland–Altman plot fitted with a linear regression line. A Bland–Altman plot provides a visual aid to assess the agreement between 2 measures [12] and the results are interpreted qualitatively. The Bland–Altman method calculates the mean difference between 2 measures and upper and lower limits of agreement based on the standard deviation of the differences between the 2 measures. If the mean difference is zero, it suggests strong agreement between the 2 measures. The Bland–Altman method requires the differences to be normally distributed; however, we found that the differences between our measures were not normally distributed, thus we followed the recommendations for nonparametric data and calculated the limits of agreement based on the 2.5th and the 97.5th percentile and display the median difference between the measures instead of the mean [13]. It should be noted that the limits of agreement are meant to be suggestive. Particularly in the case of nonparametric data, the limits of agreement tend to be wider than with the standard approach. To aid in interpretation, we include a linear regression line to the Bland–Altman plot.

## Results

### Challenges in calculating SDG Indicator 5.6.2 using current metadata

#### The effect of the weighted additive approach versus taking the mean of the 13 individual components

Taking the mean of the 13 individual components to produce the final indicator value, rather than the mean of the 4 sections, assigns greater weight to sections with a larger number of components. For example, Section IV: HIV and HPV includes 3 components related to HIV: testing and counseling (C10), treatment and care (C11), and confidentiality (C12), while Section I: Maternity Care Services contains only 1 component that reflects the entirety of maternity care (C1), despite the section’s name. The other 3 components in Section I relate to commodities (1 component) and abortion (2 components). As a result, HIV is given 3 times the weight in the indicator’s total score as a domain than is maternity care, due to the 3 components reflecting HIV versus the single component dedicated to maternity care. Similarly, Section II: Contraceptives and Family Planning has 3 components. As a result, the entire section of contraception and family planning has a 25% lower influence on the total score than the entire section of HIV and HPV, which has 4 components, as a result of unequal distribution of the number of components across the sections.

**Fig 1:**
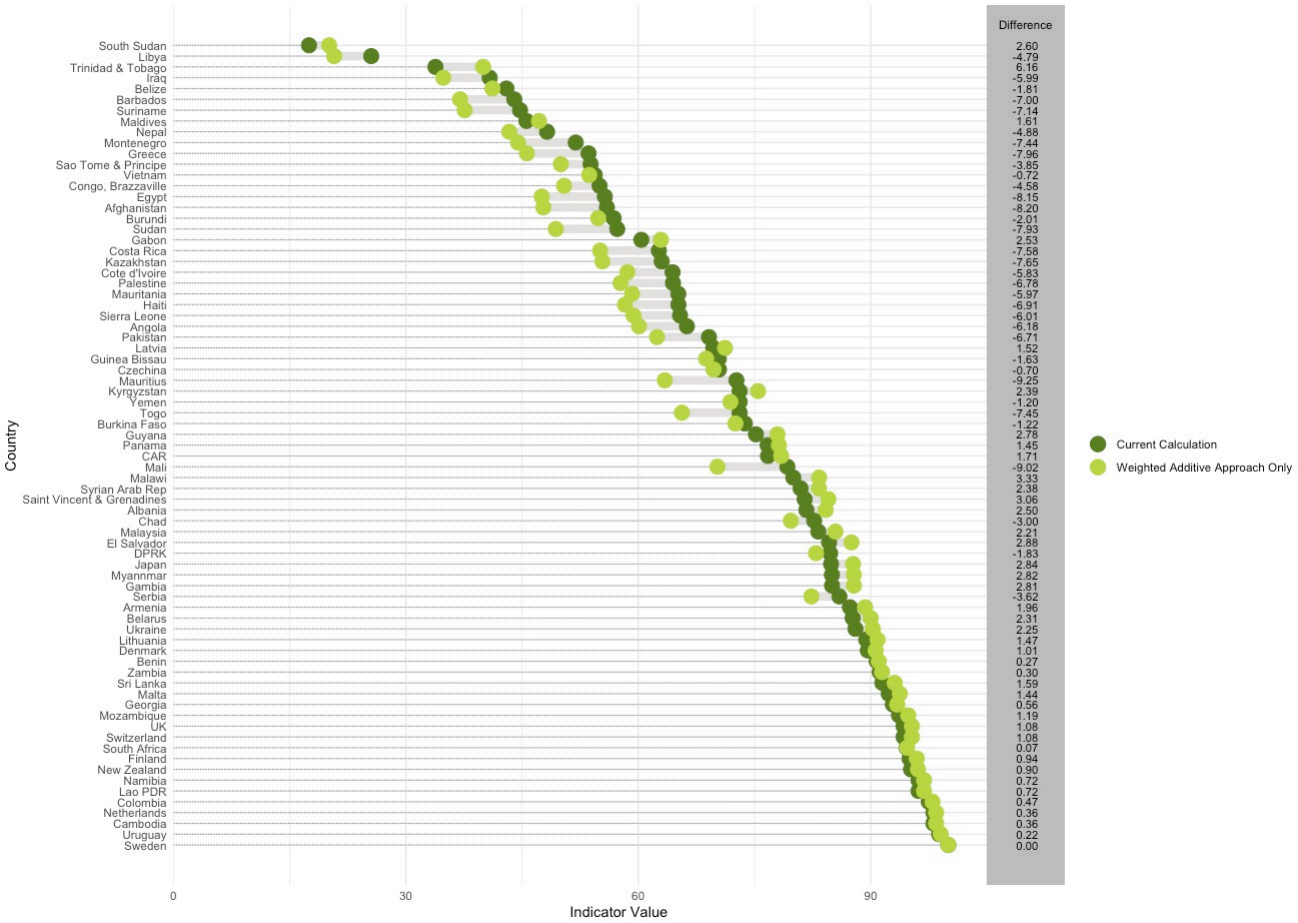
Comparison of indicator values calculated using the current metadata (taking the mean of 13 components) vs. the weighted additive approach. Note: Countries are ordered from lowest to highest indicator score following the revised approach. The dots represent the value of the indicator for each country obtained by the standard and alternative calculations. The gray lines between the dots represent the difference between the values of the indicator obtained by the standard and alternative calculations.

Giving each of the 4 sections equal weight, as opposed to weighting them based on the number of components, has a considerable impact on the indicator’s final value. Fig 1 compares the indicator value obtained for each country by taking the mean of the 13 components (using the indicator’s current metadata) to the score obtained by taking the mean of the 4 sections. Assigning equal weight to the 4 sections changed the mean score across all 75 countries by an average of −1.49 percentage points (SD = 4.03). The largest decrease in score was observed in Mauritius (−9.24 percentage points) and the largest increase in score was observed in Trinidad and Tobago (5.16 percentage points). The indicator score increased in 40 countries, decreased in 34 countries, and exhibited no change in 1 country (Sweden).

**Fig 2:**
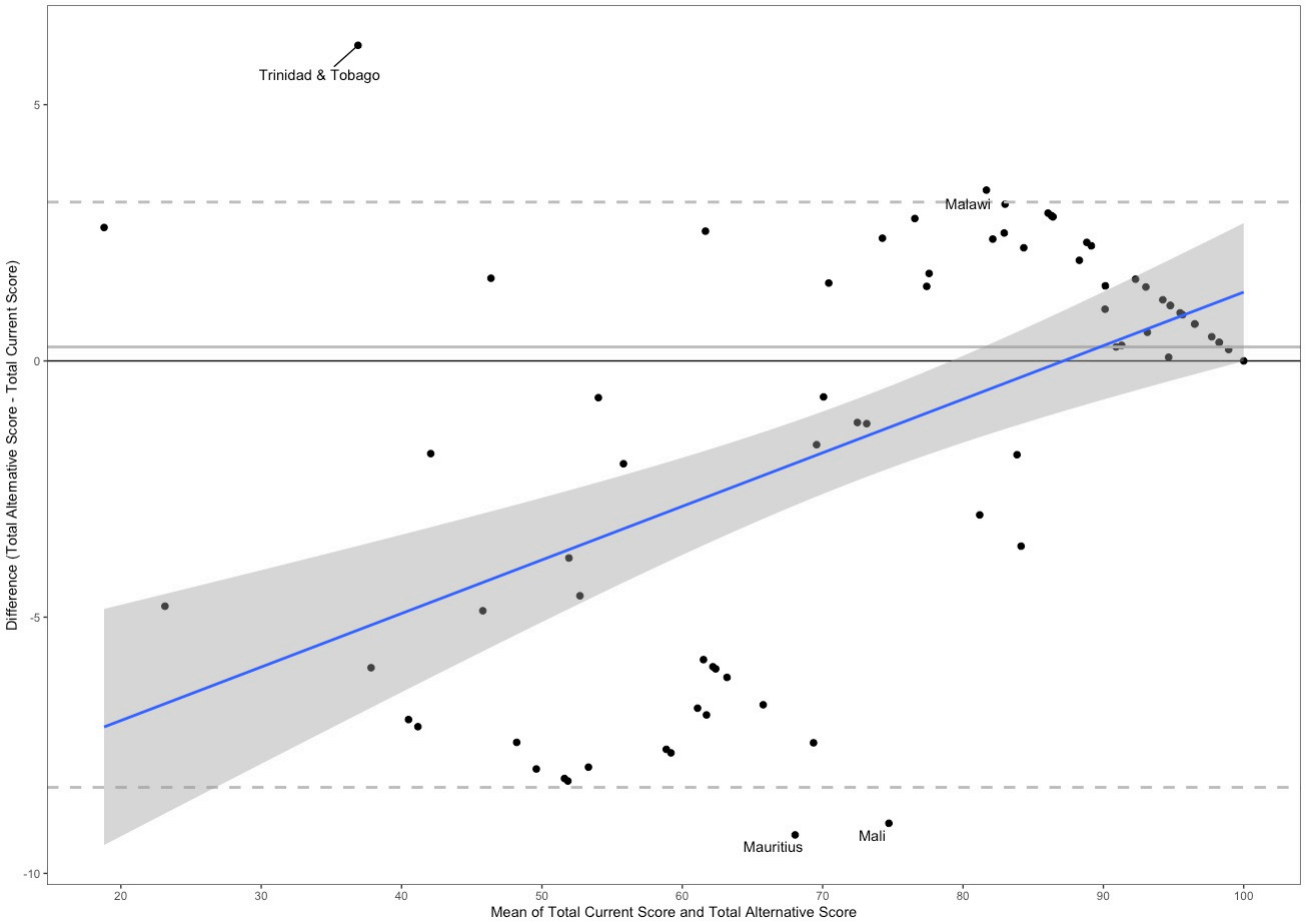
Bland–Altman plot showing the differences in section scores between the current and alternative calculation (weighted additive approach only).

A Bland–Altman plot is provided in Fig 2 comparing the current indicator value with the alternative in which only the calculation of the mean is changed. The plot indicates that on average, the alternative formula produces a slightly higher score than the current formula (median of differences = 0.27); however, the regression line suggests that the alternative formula tends to produce lower scores than the current indicator for countries with lower average scores but scores are in closer agreement for countries with higher averages scores.

#### Isolating the effect of re-expressing barriers as absent enablers

The indicator is defined so that the value of the enablers and the barriers for a given component are first calculated separately. Then, the value obtained for the barriers is subtracted from the value obtained for the enablers. Functionally, this approach assigns equal weight to both the enablers and barriers identified for a given component, regardless of their number. The weight assigned to any individual barrier is therefore an artifact of the total number of barriers identified for a given component. Further, some barriers are envisioned only to have negative consequences in the presence of an enabler, while others can have an independent effect.

For example, “Component 1: Maternity Care” has 4 barriers while “Component 13: HPV Vaccine” has 1 barrier. The barriers identified for both components include the barrier relating to whether plural legal systems exist, contradicting the enabling law. Following the current formula, the presence of a plural legal system in relation to maternity care reduces the effect of the enabling environment by 25%, while the presence of a plural legal system in relation to HPV vaccine reduces the effect of the enabling environment by 100%, given that no other barriers have been identified for HPV vaccine. This issue is illustrated in Box 3.

**Box 3: Effect of the Number of Barriers on a Component Score**

Comparing C1: Maternity Care and C13: HPV Vaccine illustrates the arbitrary nature of the effect of subtracting barriers from enablers and its impact on the interpretability of component scores.

Component 1 (C1): Maternity Care includes 1 enabler and 4 barriers:

*Enabler*

- Does the government have any laws/regulations that guarantee access to maternal health care?

*Barriers*

- If yes, are there any contradicting plural legal systems?
- Does the law include any restrictions based on age?
- Does the law include any restrictions based on marital status?
- Does the law include any restrictions based on third party authorization?

Component 13 (C13): HPV Vaccine includes 1 enabler and 1 barrier:

*Enabler*

- Does the government have any laws/policies that guarantee access to HPV vaccine for adolescent girls?

*Barrier*

- If yes, are there any contradicting plural legal systems?

Comparing Two Scenarios

In Country X, for C1 the government has a law in place that guarantees access to maternal health care; however, there are plural legal systems in place that contradict that law. No other barriers apply (e.g., there are no restrictions based on age, marital status, or third party authorization). Because there are 4 barriers and only 1 applies, ¼ of a point is subtracted from the point awarded for presence of the enabler. Thus, C1 is scored as follows, with the presence of 1 enabler and 1 barrier.

Score for C1: [(1/1)–(1/4)] = –0.75*100 = 75%

For C13, Country X has a law in place that guarantees access to HPV vaccines for adolescent girls; however, as with C1, there are also plural legal systems that contradict that law. As no other barriers are included in the score for C13, a full point is subtracted from the point that was awarded for presence of the enabler.

Score for C13: [(1/1)–(1/1)] = 0*100 = 0%

In this case, Country X is 75% of the way to achieving an optimal policy environment for C1: Maternity Care but 0% of the way to achieving an optimal policy environment for C13: HPV Vaccines. However, for each of these components, Country X has the same enabler and same barrier. In the case of C13, the presence of a plural legal system cancels out the entire effect of the enabler, while in C1, it only attenuates the effect of the enabler. The difference in score observed for each component is a simple artifact of the fact that the barriers are weighted based on their total number.

Table 2 compares the scores of “Component 1: Maternity Care” (C1) and “Component 13: HPV Vaccine (C13)” using the current and alternative formulas for Montenegro, Sudan, and Myanmar as exemplars. In Montenegro for C1, there is an enabling law present that guarantees access to maternal health care; however, there is also a plural legal system present. No other restrictions are in place. In this case, the indicator’s value results in a score of 75%, following the current metadata. In comparison, for C13: HPV Vaccine, Montenegro has a law in place that guarantees access to the HPV vaccine for adolescent girls. As with C1, there is both an enabling law and a plural legal system in place. Unlike C1, no other restrictions have been specified, thus Montenegro’s score for C13 is 0%. Re-expressing barriers positively as the absence of enablers only minimally affects the score for C1, increasing it by 5 percentage points. However, the alternative approach increases the score for C13 to 50%. The same change in score for C13 comparing the current and alternative calculation is again illustrated with data from Myanmar, given that both countries have the same barriers and enablers in place. Conversely, the alternative calculation had no impact on Myanmar’s score for C1, which received a score of 100% using both the current and alternative formulas, because there are no barriers in place for C1 in Myanmar, unlike in Montenegro.

**Table 2:** Comparison of current and alternative calculation of indicator component scores for C1 (Maternity Care) and C13 (HPV Vaccine) in selected countries.

Country	Component	Enabling Law	Plural Legal System	Restrictions			Current Score	Alternative Score
				Age	Marital Status	Third Party Authorization		
Montenegro	C1: Maternity Care	Yes	Yes	No	No	No	75%	80%
	C13: HPV Vaccine	Yes	Yes	-	-	-	0%	50%
Myanmar	C1: Maternity Care	Yes	No	No	No	No	100%	100%
	C13: HPV Vaccine	Yes	Yes	-	-	-	0%	50%
Sudan	C1: Maternity Care	Yes	Yes	Yes	Yes	Yes	0%	20%
	C13: HPV Vaccine	Yes	Yes	-	-	-	0%	50%

Sudan’s data provided in Table 2 illustrates a different challenge that results from subtracting barriers from enablers. For C1, Sudan has an enabling law present that guarantees access to maternity care. At the same time, there is a plural legal system in place that contradicts the law, as well as restrictions based on age, marital status, and third party authorization. As the current scoring system weights the barriers based on their number (which in this case assigns them each a weight of 0.25 percentage points because there are 4), Sudan’s score for C1 is 0%. Similarly, for C13, Sudan has an enabling law present that guarantees access to the HPV vaccine for adolescent girls; however, there is also a plural legal system in place that contradicts that law. In this case, as restrictions relating to age, marital status, and third party authorization have not been specified as barriers in the indicator’s metadata, the only barrier identified is assigned a value of a full point. In other words, the presence of a plural legal system for C1 detracts 25% of the progress reflected by the presence of the enabling law, whereas for C13, the presence of a plural legal system negates 100% of the progress reflected by the enabling law. Re-expressing barriers as absent enablers and taking the mean of the items in each component to calculate the score results in a score of 20% for C1 and 50% for C2 for Sudan.

**Fig 3:**
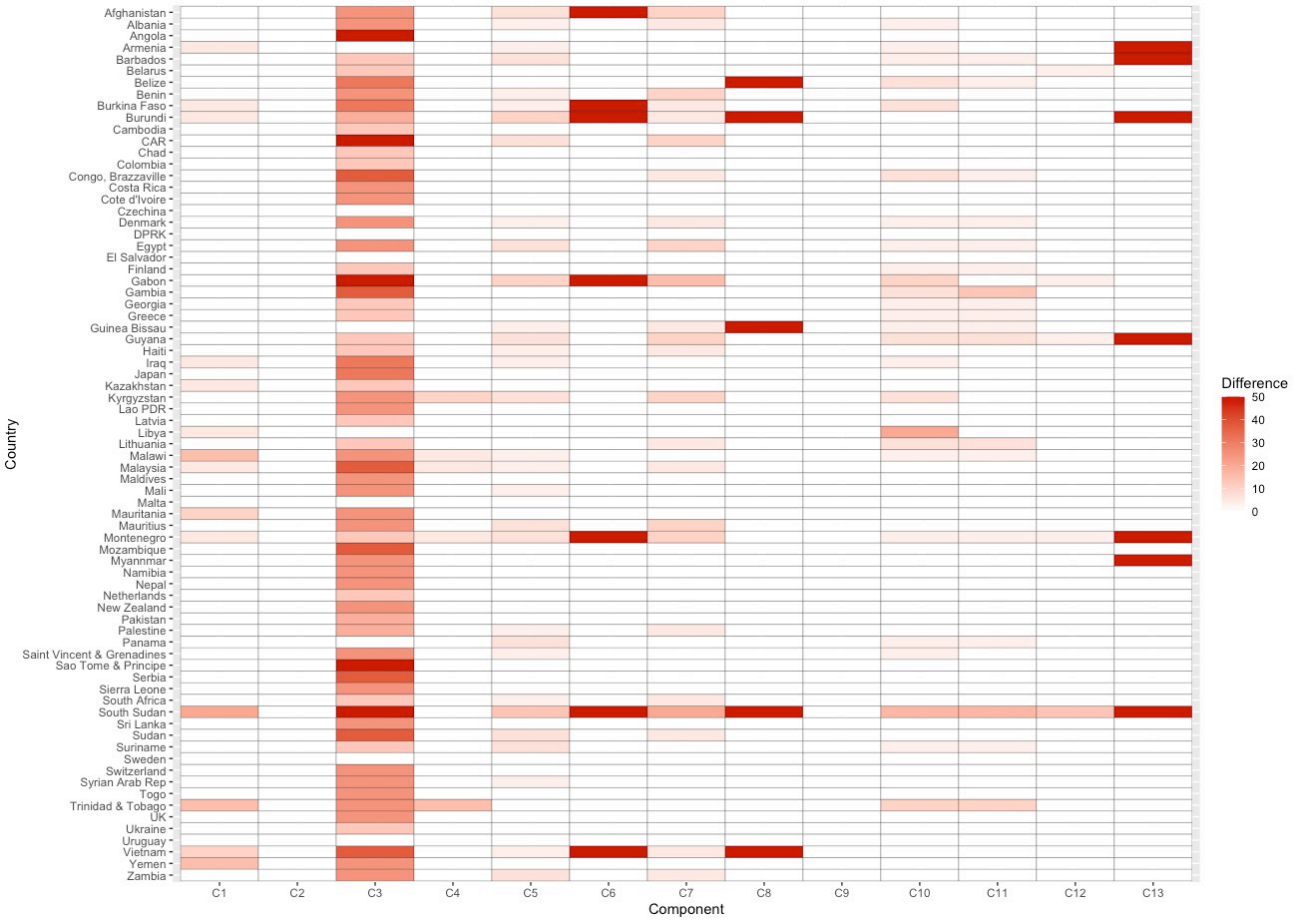
Difference in component scores after re-expressing barriers as absent enablers. Note: Countries are ordered in alphabetical order. Difference refers to the difference in component score obtained comparing the standard with the alternative calculation of the indicator.

Fig 3 shows the change in individual component scores after re-expressing barriers as absent enablers. Component scores did not change for C2: Life Saving Commodities and C9: Sexuality Education Curriculum Topics as no barriers were identified for those components in the indicator’s metadata.

Re-expressing barriers as the absence of enablers has the most substantial impact on scores for “Component 3: Abortion.” The score for “Component 3: Abortion” changed for all countries except those with a score of 100% per the indicator’s current formulation. The value of “Component 3: Abortion” increased by 50 percentage points in 5 countries (Angola, Central African Republic, Gabon, Sao Tome and Principe, and South Sudan), which was the largest increase observed in the component score after re-expressing barriers as absent enablers. In these countries, abortion is permitted under the 4 legal grounds specified in the indicator, but all 3 of the restrictions specified are also present in the country’s legal framework. Under the previous scoring, these countries would have received a score of zero for the component, alongside those countries in which abortion is not legal on any of the specified legal grounds.

Table B in S1 Text summarizes the legal grounds on which abortion is legal, the barriers in place, and each country’s score for “Component 3: Abortion” comparing the current formula’s component score with the component score obtained after re-expressing barriers as the absence of enablers. The largest differences in the 2 scoring approaches for “Component 3: Abortion” were observed among the countries with the lowest scores following the indicator’s current computation. Following the current formula, the 16 countries with a score of 0 have between 0 and 4 legal grounds present that enable women to legally obtain an abortion, and the number of barriers present either outweighs the number of legal grounds or equals them. Among these countries, there is inconsistency as to whether women can be criminally charged for illegal abortion. After re-expressing barriers as absent enablers, only 4 countries have a score of zero. All 4 of these countries have no legal grounds on which abortion is legal and also have the added barrier that women can be criminally charged for illegal abortion. Two countries have a score of 12.5%, in which abortion is not legal on any grounds; however, in these countries, women cannot be criminally charged for an illegal abortion. In comparison, countries that have a score between 6.25% and 12.5% for “Component 3: Abortion” following the current calculation method, have between 1 and 2 legal grounds present, but the total number of barriers (including the application of criminal charges) outweigh the enablers by either 2 to obtain a score of 6.25% or 1 to obtain a score of 12.5%.

In general, as scores for “Component 3: Abortion” obtained using the current formula increase, there is less variation in the underlying data. All countries that received a score of 50% using the current metadata have all 4 legal grounds present and 2 barriers present. Therefore, a score of 50% obtained from the indicator’s current formula indicates that a country has 2 more enablers present than barriers. After re-expressing barriers as the absence of enablers, we find that all countries receiving a score of 50% have at least 1 legal ground present, and that there is an equal number of barriers and enablers present. Under both indicator formulations, no changes were observed among the 6 countries that received a score of 100%. In all of these countries, abortion is legal on all 4 grounds and there are no barriers in place.

After re-expressing barriers as absent enablers, scores for “Component 6: Contraceptive Consent,” “Component 8: Sexuality Education Curriculum Laws,” and “Component 13: HPV Vaccine” either increased by 50 percentage points or did not change at all. A similar characteristic among these components is that only 1 barrier was identified in the current metadata. In countries where both the one enabler identified and the one barrier identified were present, re-expressing the barriers as enablers attenuated the effect of the enabler rather than negating its effect entirely, thus increasing the scores in those countries by 50 percentage points.

The remaining components showed more granular changes after re-expressing barriers as enablers. These components were characterized by multiple barriers, thus attenuating the effect of any individual barrier. For example, South Sudan had an enabling law present for “Component 1: Maternity Care” but also had the 4 identified barriers present in the country’s legal framework. As a result of re-expressing barriers as enablers, South Sudan’s score for “Component 1: Maternity Care” increased from the value of 0 to 20 percentage points—thereby distinguishing it from other countries, such as Sao Tome and Principe, which do not have an enabling law present.

**Fig 4:**
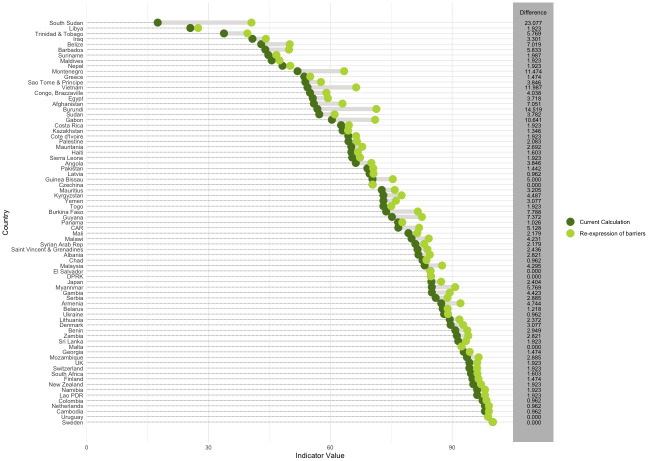
Comparison of indicator values calculated using the current metadata vs. the score obtained from re-expressing barriers as absent enablers. Note: Countries are ordered from lowest to highest indicator score following the revised approach. The different colored dots represent the value of the indicator for each country obtained by the standard and alternative calculations. The gray lines between the dots represent the magnitude of the difference between the values of the indicator obtained by the standard and alternative calculations.

**Fig 5:**
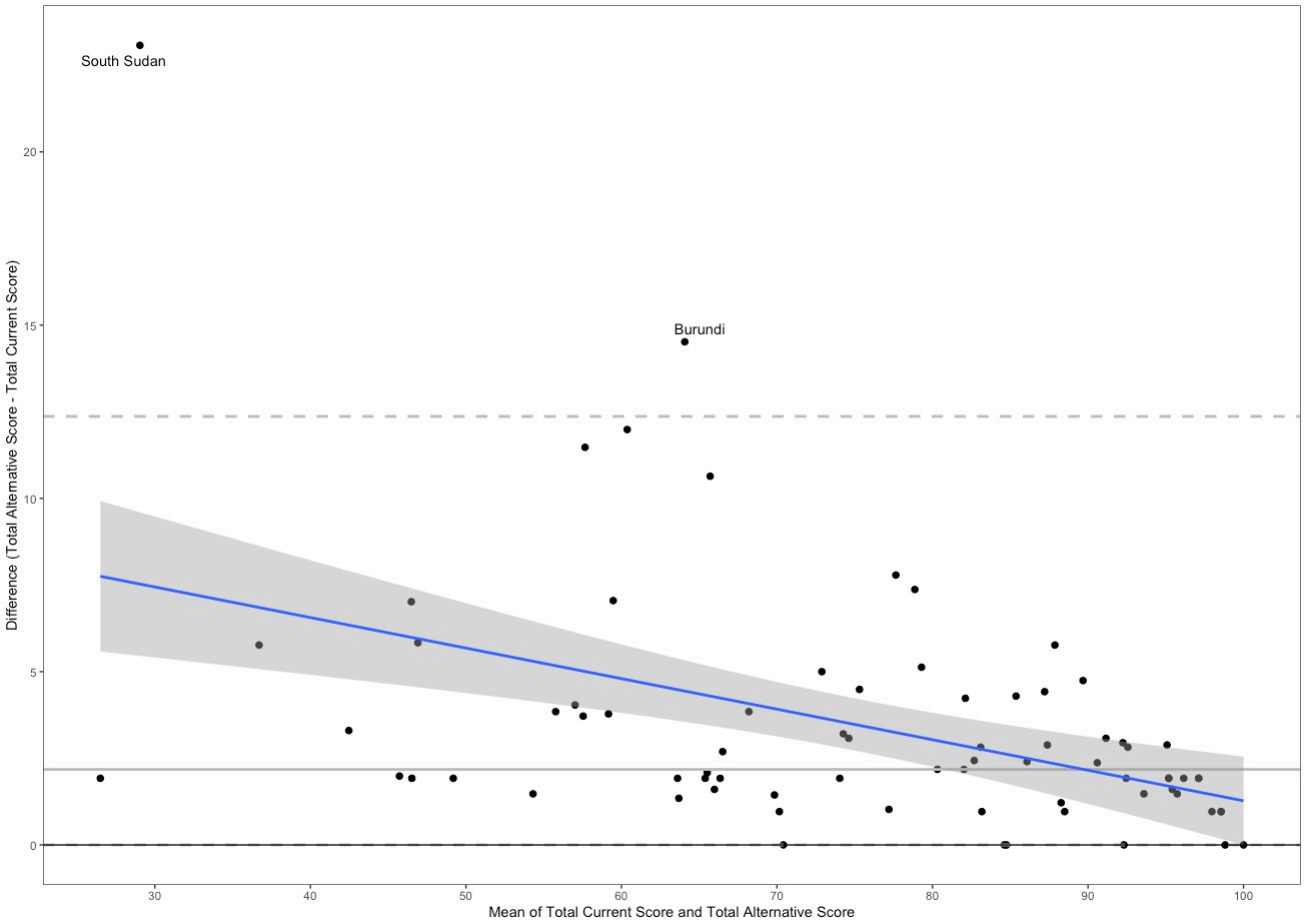
Bland–Altman plot showing the differences in section scores between the current and alternative calculation (re-expression of barriers as absent enablers only).

On average, re-expressing barriers as enablers increased the value of the indicator by 3.44 percentage points. Six countries saw no change in the value of the indicator (Czechia, El Salvador, Democratic People’s Republic of Korea, Malta, Uruguay, and Sweden) after re-expressing barriers as enablers. The largest change in score occurred in South Sudan, with an increase of 23.07 percentage points. Fig 4 shows the difference in the total value of the indicator after re-expressing barriers as absent enablers for each country, but without taking the mean of the 13 components rather than the 4 sections in order to isolate the change’s effect. Fig 5 provides the Bland–Altman plot to further compare the difference between the 2 formulations of the indicator. The Bland–Altman plot for this change suggests that this change produces a significant change to the score as the limits of agreement lie above the value zero. Similar to the effect of isolating the changes to the calculation of the mean, there tends to be better agreement between the 2 measures for countries with higher average scores.

### Comparison of total scores for SDG Indicator 5.6.2 derived from the current versus alternative formula

**Fig 6:**
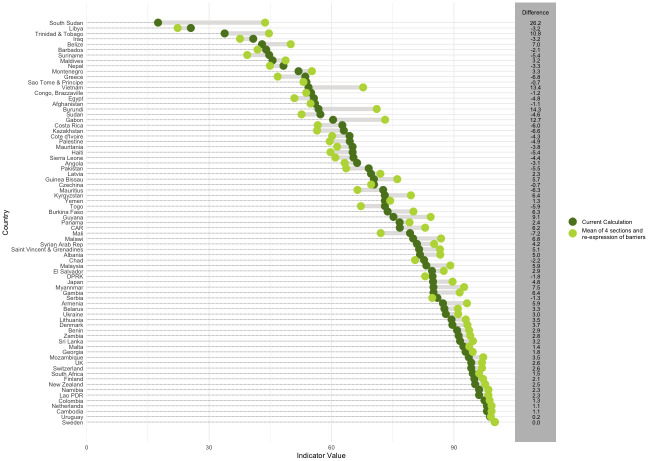
Differences in indicator value between the current and proposed calculation of SDG Indicator 5.6.2 by country. Note: Countries are ordered from lowest to highest indicator score following the revised approach. The different colored dots represent the value of the indicator for each country obtained by the standard and alternative calculations. The gray lines between the dots represent the magnitude of the difference between the values of the indicator obtained by the standard and alternative calculations.

Fig 6 presents the score for SDG Indicator 5.6.2 obtained from the current formula, the alternative formula (in which both the weighted additive approach and the re-expression of barriers as absent enablers are applied), and the difference for each country. Differences in the total indicator between the alternative versus the current metadata score range from −7.18 percentage points in Mali to 26.21 percentage points in South Sudan. The majority of countries (*n* = 47) had an increase in total indicator score as a result of the alternative formula, while 27 countries had a decrease in score. Only 1 country, Sweden, saw no change in score as it scored 100% under both rubrics. Fig A in S1 Text shows the difference in score for each section between the old and the new calculations. The magnitude of change between the 2 formulations was considerable, with 15 countries seeing a change greater than 10 percent.

**Fig 7:**
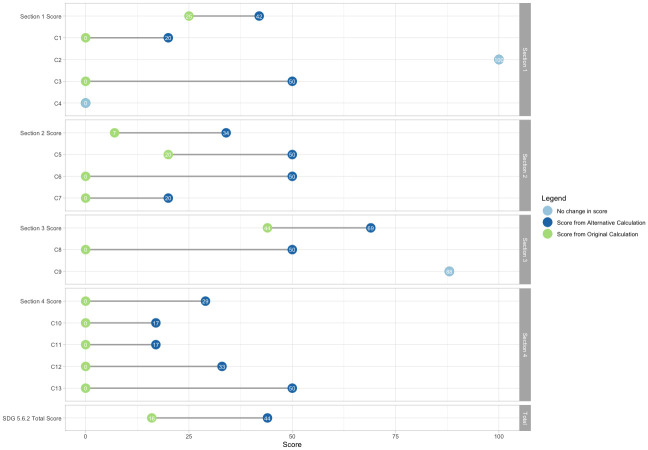
Differences in section and component scores between the current and proposed formula for SDG Indicator 5.6.2 in South Sudan. Note: The dots represent the value of the indicator for each country obtained by the standard and alternative calculations. The gray lines between the dots represent the difference between the values of the indicator obtained by the standard and alternative calculations.

South Sudan had the greatest change in score observed out of all countries. Fig 7 provides a detailed visualization of how South Sudan’s score for each section and its components change using the alternative indicator calculation. Of note, scores for Sections 2, 3, and 4 increased considerably for South Sudan as a result of the alternative formulation. For all 3 sections, the change was driven by components in which only 1 enabler was identified, specifically “Component 6: Contraceptive Consent (C6),” “Component 8: Sexuality Education Curriculum Laws (C8),” and “Component 13: HPV Vaccine (C13).” Using the current formula, the existence of a single barrier erases the entire positive effect of the enabling law present for each of these components, leaving Sudan with a score of 0% for each one. Instead, the alternative formula results in a score of 50% for these components, which distinguishes South Sudan’s policy environment—where there is an enabling law present but also the presence of a plural legal system that acts as a barrier—from countries in which there is no enabling law present.

**Fig 8:**
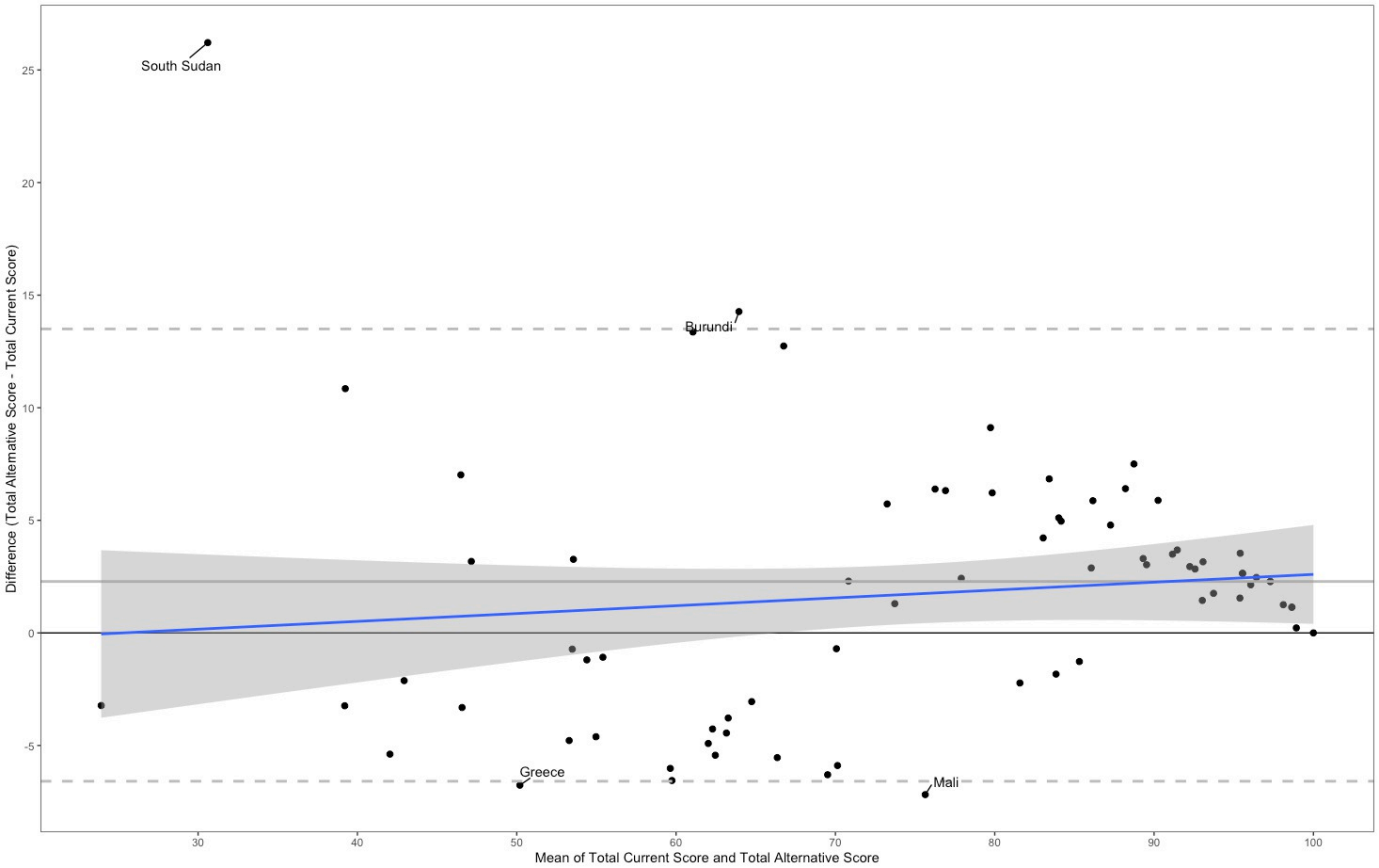
Bland–Altman plot showing the differences in section scores between the current and alternative calculation of SDG Indicator 5.6.2 by country.

A Bland–Altman plot comparing the performance of the current to the proposed indicator calculation is presented in Fig 8. The mean difference between the scores produced by the 2 measures is 2.28 suggesting that the 2 methods may produce systematically different results. Further, the variability in the differences between scores plotted is not consistent as the mean changes, and there is visible fluctuation. The spearman rank correlation coefficient between the mean and the difference of the measures is 0.25, *p*-value = 0.34. The Bland–Altman plot suggests that the alternative formulation produces lower values than the current calculation of the indicator for countries with a lower average score but higher values for countries with higher average scores. To examine the differences in the 2 scores by section, Fig B in S1 Text provides a Bland–Altman plot comparing the total score for each individual section produced by the 2 measures. While the scores for all sections generally indicate that the measures are less concordant at lower mean scores, the plots indicate that the alternative formula produces significantly higher total scores for sections and 2 and 4 given that the limits of agreement do not include the value of zero.

## Discussion

We analyzed secondary data to calculate SDG Indicator 5.6.2 using a proposed revision to the formula that addresses stakeholder calls to address specific concerns in the way the indicator is currently calculated. By examining the convergent validity between the current indicator and the alternative calculation of the measure, we find inconsistencies that point to underlying differences in how the indicator is operationalized that have meaningful implications for programs and policies. These changes are particularly relevant given ongoing discussions about how to revise measures for the post-SDG era. While there is no gold standard for this indicator with which to compare its measurement, the fact that 2 measurement approaches for the same underlying construct produce systematically different values points to a need to revisit the formulation of the existing indicator. Taking our results together, the differences in indicator values appear to be concentrated among countries that have the lowest scores using the current computational method, with less variation as scores increase. Our proposed revisions may improve the overall stability and interpretability of the indicator’s value across countries, especially for countries that have the most still to achieve.

At the component level, differences in scores observed between the 2 indicators result in substantively different interpretations of progress made at the country level, especially in the case of Component 3: Abortion. In the current formulation, barriers are given equal weight to enablers, which are then subtracted from the enablers as they are envisioned to undermine the policy environment. However, the results show the wide variation in the legality of abortion in countries that received a score of zero for Component 3. In some countries with a score of zero, abortion is legal on all 4 legal grounds while in others it is not legal on any of the grounds specified. Simply by re-expressing barriers positively as the absence of enablers, the only countries that receive a score of 0% are those in which abortion is not legal on any of the grounds specified and women can additionally be criminally charged for an illegal abortion. If the indicator’s value is to be interpreted as the percentage to which a country has achieved the enabling laws and regulations for full and equal access for women and men aged 15 years and older to sexual and reproductive care, education, and information, it is difficult to make the case that a country in which abortion is not legal on any grounds has achieved the same degree of progress as one in which it is legal on all 4 legal grounds specified regardless of the number barriers put into place—yet under the current indicator, such countries could both receive a score suggesting that zero progress has been made in either country.

At the country level, the changes proposed to the indicator’s calculation suggest different priorities for action to drive progress in achieving full and equal access to sexual and reproductive health care, information, and education. Drawing on the results for South Sudan as an example, the current indicator gives South Sudan a score of 0% for 11 of the 13 components, suggesting no progress has been made, despite there being an enabling law present in 10 of the 11 components. The alternative formulation leaves only 1 component (“Component 4: Post-Abortion Care”) with a score of zero, where there is no enabling law present. We believe that these differences in the policy environment are meaningful, and that suggesting zero progress has been made to both by assigning them the same score does not accurately reflect the policy landscape. As a result of the alternative measurement approach, Sudan’s score increased from 16% to 44%, representing a significant increase in the representation of progress that has been made. Policymakers can use the indicator to more readily understand areas where there are no laws in place (as in post abortion care) or areas where there has been some progress, but substantial barriers are barriers are present (low scoring components) to identify future opportunities for investment.

Our revisions also make the indicator more similar in formulation to other global indicators. The weighted additive approach we use to emphasize the substantive domain (section) over each individual item in the total score is consistent with the calculation of other similar indices [13–15]. We are not aware of any rationale that has been provided that justifies giving some of the substantive domains included in the indicator’s metadata more importance in the final score than other substantive domains, as is done by taking the mean of the 13 components as specified in the indicator’s current formulation. In fact, the current approach may overstate progress because we observe that the total score for most countries decreases using the proposed alternative scoring compared to the total score generated from the current formulation. Further, no other SGD indicator, or other global indicator of which we are aware, follows an approach by which elements envisioned to undermine other elements are subtracted. As illustrated by our results, we believe that this aspect of the indicator negatively affects its construct validity.

Our study reflects certain strengths. It presents a critical appraisal of a key global indicator for gender empowerment and equality using real country data while proposing a simple, actionable solution to improve its validity. The results and implications for practice are globally relevant due to this indicator’s position in the SDG monitoring framework.

In terms of limitations, this work focuses solely on developing an alternative formula to calculate the value of the indicator. Given that there is no gold standard for how to measure the underlying construct related to SDG 5.6.2, we rely on comparing 2 proxy measures: we compare the proxy that is currently in use and widely accepted to a new proxy measurement reconfigured to measure the same underlying construct. While our results point to potential problems with the construct validity of the current indicator (i.e., how effectively and accurately operationalization of the measure reflects the underlying concepts it intends to capture), we cannot specify which indicator performs better as there is no gold standard for measurement. Future research may consider examining the associations between the indicator and other related indicators, such as maternal mortality or adolescent pregnancy, to further assess construct validity of the indicator. Further, assessing content validity (i.e., how well the component parts reflect the universe of phenomena it intends to capture) is outside the scope of this study. Such validity rests on the strength of the evidence that drove selection of the sections, the component enablers and barriers, and the rigor of the process to determine their comprehensiveness. Here, we only explored measure performance, without altering the underlying content.

While assessing content validity of the indicator was outside the scope of this study, our results nonetheless highlight several related concerns worth mentioning. We argue the need for close examination related to the functioning of enablers and barriers as specified in the indicator’s current formulation, as the current formulation of the indicator is not robust to change if new barriers or enablers are identified. For example, although the indicator’s metadata note that applicable legal indicators were not identified during the indicator development process for C2: Life-saving Commodities and C9: Sexuality Education Curriculum Topics, one could envision applicable legal barriers for these components. Similarly, for “Component 13: HPV Vaccine,” only 1 barrier was identified related to plural legal systems. However, the applicability of restrictions based on age, sex, marital status, or third party authorization could easily be envisioned with regard to the HPV vaccine, just as these same barriers were deemed to be relevant to “Component 12: HIV Confidentiality.” Our study demonstrates that the scoring of this component would change fundamentally should such barriers be added in the future to “Component 13: HPV Vaccine,” and should be carefully examined in future iterations of the indicator. Similarly, another threat to the indicator’s content validity related to the equal weighting of enablers and barriers is that it does not allow for nuance. For example, weighting barriers and enablers equally in “Component 3: Abortion” does not capture whether a barrier applies to all or some of the enablers identified. For example, a law may require a husband’s consent for a woman to obtain an abortion in the case of fetal impairment, but the husband’s consent may not be required to save a woman’s life. As this concern is related to the content validity of the indicator, addressing it is beyond the scope of our study, but it should be considered in future revisions of the indicator.

Some experts have argued that the decision to weight barriers and enablers equally was rooted in a rights-based approach focused on equity, contending that the presence of a positive law or regulation, if it is contradicted by plural legal systems or restrictions, does not guarantee such access to everyone, because plural legal systems may differentially restrict access based on specific characteristics (e.g., geography, sex). While we understand the rationale behind the decision, we believe that the challenges introduced by this approach undermine its overall validity.

In conclusion, simple changes to the calculation of SDG Indicator 5.6.2 now or in the post-SDG period would arguably result in a more meaningful measure of the policy environment related to sexual and reproductive health and rights. Future work should target systematic evaluation of the content of this indicator with an aim of improving this measure for future iterations [9].

## Supporting information

S1 Text Table A. Documentation for current SDG Indicator 5.6.2 scoring and alternative calculation. Table B. Comparison of Scores for “Component 3: Abortion” (Current versus Proposed Calculation) for 75 Countries. Fig A. Differences in section scores between the current and proposed calculation of SDG Indicator 5.6.2 by country. Fig B. Bland–Altman plot showing the differences in section scores between the current and alternative calculation of SDG Indicator 5.6.2 by country by section.
(DOCX)

## Abbreviations

EPMM: Strategies toward Ending Preventable Maternal Mortality
ICPD: International Conference on Population and Development
IRB: Institutional Review Board
SDG: Sustainable Development Goal
