# Supplementary material for: Comparative analysis of 2 approaches to monitor countries’ progress towards full and equal access to sexual and reproductive health care, information, and education in 75 countries: An observational validation study
Source: PLoS Med. 2024 Dec 31;21(12):e1004476. doi: 10.1371/journal.pmed.1004476 (PMC11687741; doi:10.1371/journal.pmed.1004476)
Supplement: S1 Text — Table A. Documentation for current SDG Indicator 5.6.2 scoring and alternative calculation. Table B. Comparison of Scores for “Component 3: Abortion” (Current versus Proposed Calculation) for 75 Countries. Fig A. Differences in section scores between the current and proposed calculation of SDG Indicator 5.6.2 by country. Fig B. Bland–Altman plot showing the differences in section scores between the current and alternative calculation of SDG Indicator 5.6.2 by country by section. (DOCX) [file pmed.1004476.s001.docx]

**S1 Supporting Information**

“Comparative analysis of two approaches to monitor countries’ progress towards full and equal access to sexual and reproductive health care, information, and education in 75 countries: An Observational Validation Study

**Documentation for current SDG Indicator 5.6.2 scoring**

Individual questions in the scoring matrix are specified as follows and obtained from the United Nations 12^th^ Inquiry among Governments on Population and Development, Module II: Fertility, Family Planning and Reproductive Health (available: <https://www.un.org/development/desa/pd/themes/population-policies/inquiry12>).

Table A.

|  |  | **Current Specification of Enablers and Barriers** | | | | | |
| --- | --- | --- | --- | --- | --- | --- | --- |
| **Section** | **Components** | **Enablers** | | | **Barriers** | | |
|  |  | Quest# | Score  e_i_ | E_i_ | Quest# | Score  b_i_ | B_i_ |
| S1: Section 1 | C1: Maternity Care | 2.18 |  | 1 | 2.19 |  | 4 |
|  |  |  |  |  | 2.20_a |  |  |
|  |  |  |  |  | 2.20_b |  |  |
|  |  |  |  |  | 2.20_c |  |  |
|  | C2: Life Saving Commodities | 2.22_a |  | 13 |  |  | 0 |
|  |  | 2.22_b |  |  |  |  |  |
|  |  | 2.22_c |  |  |  |  |  |
|  |  | 2.22_d |  |  |  |  |  |
|  |  | 2.22_e |  |  |  |  |  |
|  |  | 2.22_f |  |  |  |  |  |
|  |  | 2.22_g |  |  |  |  |  |
|  |  | 2.22_h |  |  |  |  |  |
|  |  | 2.22_i |  |  |  |  |  |
|  |  | 2.22_j |  |  |  |  |  |
|  |  | 2.22_k |  |  |  |  |  |
|  |  | 2.22_l |  |  |  |  |  |
|  |  | 2.22_m |  |  |  |  |  |
|  | C3: Abortion | 2.34_a |  | 4 | 2.35_b |  | 4 |
|  |  | 2.34_b |  |  | 2.35_d |  |  |
|  |  | 2.34_d |  |  | 2.35_e |  |  |
|  |  | 2.34_f |  |  | 2.36 |  |  |
|  | C4: Post-Abortion Care | 2.37 |  | 1 | 2.38 |  | 4 |
|  |  |  |  |  | 2.39_a |  |  |
|  |  |  |  |  | 2.39_b |  |  |
|  |  |  |  |  | 2.39_c |  |  |
| S2: Section 2 | C5: Contraceptive Services | 2.23_a |  | 1 | 2.24_a |  | 5 |
|  |  |  |  |  | 2.26_a_1 |  |  |
|  |  |  |  |  | 2.26_a_2 |  |  |
|  |  |  |  |  | 2.26_a_3 |  |  |
|  |  |  |  |  | 2.26_a_4 |  |  |
|  | C6: Contraceptive Consent | 2.23_c |  | 1 | 2.24_c |  | 1 |
|  | C7: Emergency Contraception | 2.23_b |  | 1 | 2.24_b |  | 4 |
|  |  |  |  |  | 2.26_b_1 |  |  |
|  |  |  |  |  | 2.26_b_2 |  |  |
|  |  |  |  |  | 2.26_b_3 |  |  |
| S3: Section 3 | C8: Sexuality Education Curriculum Laws | 2.15 |  | 1 | 2.16 |  | 1 |
|  | C9: Sexuality Education Curriculum Topics | 2.17_a |  | 8 |  |  | 0 |
|  |  | 2.17_b |  |  |  |  |  |
|  |  | 2.17_c |  |  |  |  |  |
|  |  | 2.17_d |  |  |  |  |  |
|  |  | 2.17_e |  |  |  |  |  |
|  |  | 2.17_f |  |  |  |  |  |
|  |  | 2.17_g |  |  |  |  |  |
|  |  | 2.17_h |  |  |  |  |  |
| S4: Section 4 | C10: HIV Counselling and Test Services | 2.30_a |  | 1 | 2.31_a |  | 5 |
|  |  |  |  |  | 2.32_a_1 |  |  |
|  |  |  |  |  | 2.32_a_2 |  |  |
|  |  |  |  |  | 2.32_a_3 |  |  |
|  |  |  |  |  | 2.32_a_4 |  |  |
|  | C11: HIV Treatment and Care Services | 2.30_b |  | 1 | 2.31_b |  | 5 |
|  |  |  |  |  | 2.32_b_1 |  |  |
|  |  |  |  |  | 2.32_b_2 |  |  |
|  |  |  |  |  | 2.32_b_3 |  |  |
|  |  |  |  |  | 2.32_b_4 |  |  |
|  | C12: HIV Confidentiality | 2.30_c |  | 1 | 2.31_c |  | 0 |
|  |  |  |  |  | 2.32_c_1 |  |  |
|  |  |  |  |  | 2.32_c_2 |  |  |
|  |  |  |  |  | 2.32_c_3 |  |  |
|  |  |  |  |  | 2.32_c_4 |  |  |
|  | C13: HPV Vaccine | 2.13 |  | 1 | 2.14 |  | 1 |

Table B: Comparison of Scores for “Component 3: Abortion” (Current versus Proposed Calculation) for 75 Countries

| **Proposed Calculation** | | | | | | **Current Calculation** | | | | | |
| --- | --- | --- | --- | --- | --- | --- | --- | --- | --- | --- | --- |
| **Country** | **C3 Score** | **# of Legal grounds** | **# of Barriers** | **Criminal Charges Apply** | **Summary** | **Country** | **C3 Score** | **# of Legal grounds** | **# of Barriers** | **Criminal Charges Apply** | **Summary** |
| El Salvador | 0 | 0 | NA | 1 | No legal grounds present, women can be criminally charged | Angola | 0 | 4 | 3 | 1 | Between 0 and 4 legal grounds present. Barriers either outweigh enablers or equal enablers. |
| Libya | 0 | 0 | NA | 1 |  | CAR | 0 | 4 | 3 | 1 |  |
| Malta | 0 | 0 | NA | 1 |  | Congo, Brazzaville | 0 | 3 | 3 | 1 |  |
| Panama | 0 | 0 | NA | 1 |  | Egypt | 0 | 2 | 3 | 1 |  |
| Haiti | 0.125 | 0 | NA | 0 | No legal grounds present; No criminal charges for women obtaining an illegal abortion | El Salvador | 0 | 0 | NA | 1 |  |
| Suriname | 0.125 | 0 | NA | 0 |  | Gabon | 0 | 4 | 3 | 1 |  |
| Egypt | 0.25 | 2 | 3 | 1 | At least one legal ground present. Number of barriers outweigh enablers by 2 | Haiti | 0 | 0 | NA | 0 |  |
| Malawi | 0.25 | 2 | 3 | 1 |  | Libya | 0 | 0 | NA | 1 |  |
| Pakistan | 0.25 | 1 | 2 | 1 |  | Malawi | 0 | 2 | 3 | 1 |  |
| Palestine | 0.25 | 1 | 3 | 0 |  | Malaysia | 0 | 3 | 3 | 1 |  |
| Syrian Arab Rep | 0.25 | 2 | 3 | 1 |  | Malta | 0 | 0 | NA | 1 |  |
| Congo, Brazzaville | 0.375 | 3 | 3 | 1 | At least one legal ground present. Number of barriers outweigh enablers by 1 | Panama | 0 | 0 | NA | 1 |  |
| Malaysia | 0.375 | 3 | 3 | 1 |  | Sao Tome & Principe | 0 | 4 | 3 | 1 |  |
| Mauritania | 0.375 | 1 | 2 | 0 |  | South Sudan | 0 | 4 | 3 | 1 |  |
| Myanmar | 0.375 | 1 | 1 | 1 |  | Suriname | 0 | 0 | NA | 0 |  |
| Sri Lanka | 0.375 | 1 | 2 | 0 |  | Syrian Arab Rep | 0 | 2 | 3 | 1 |  |
| Afghanistan | 0.5 | 2 | 2 | 0 | At least one legal ground present; barriers are equal to the number of enablers | Pakistan | 6.25 | 1 | 2 | 1 | Barriers outweigh enablers by 2 |
| Angola | 0.5 | 4 | 3 | 1 |  | Palestine | 6.25 | 1 | 3 | 0 |  |
| Belize | 0.5 | 3 | 2 | 1 |  | Mauritania | 12.5 | 1 | 2 | 0 | Barriers outweigh enablers by 1 |
| Burkina Faso | 0.5 | 3 | 2 | 1 |  | Myanmar | 12.5 | 1 | 1 | 1 |  |
| CAR | 0.5 | 4 | 3 | 1 |  | Sri Lanka | 12.5 | 1 | 2 | 0 |  |
| Gabon | 0.5 | 4 | 3 | 1 |  | Belize | 18.75 | 3 | 2 | 1 | Ratio of enablers to barriers is either 3:3 or 1:1 |
| Iraq | 0.5 | 1 | 1 | 0 |  | Burkina Faso | 18.75 | 3 | 2 | 1 |  |
| Japan | 0.5 | 3 | 2 | 1 |  | Iraq | 18.75 | 1 | 1 | 0 |  |
| Sao Tome & Principe | 0.5 | 4 | 3 | 1 |  | Japan | 18.75 | 3 | 2 | 1 |  |
| South Sudan | 0.5 | 4 | 3 | 1 |  | Afghanistan | 25 | 2 | 2 | 0 | Ratio of enablers to barriers is either 2:2 or 4:3 |
| Switzerland | 0.5 | 2 | 1 | 1 |  | Gambia | 25 | 4 | 2 | 1 |  |
| Trinidad & Tobago | 0.5 | 2 | 1 | 1 |  | Mozambique | 25 | 4 | 2 | 1 |  |
| UK | 0.5 | 2 | 2 | 0 |  | Serbia | 25 | 4 | 2 | 1 |  |
| Yemen | 0.5 | 2 | 2 | 0 |  | Sudan | 25 | 1 | 0 | 0 |  |
| Costa Rica | 0.625 | 2 | 0 | 1 | At least one legal ground present; enablers outweigh barriers by 1 | Switzerland | 25 | 2 | 1 | 1 |  |
| Cote d'Ivoire | 0.625 | 3 | 1 | 1 |  | Trinidad & Tobago | 25 | 2 | 1 | 1 |  |
| Gambia | 0.625 | 4 | 2 | 1 |  | UK | 25 | 2 | 2 | 0 |  |
| Maldives | 0.625 | 3 | 1 | 1 |  | Vietnam | 25 | 4 | 3 | 0 |  |
| Mozambique | 0.625 | 4 | 2 | 1 |  | Yemen | 25 | 2 | 2 | 0 |  |
| New Zealand | 0.625 | 3 | 1 | 1 |  | Costa Rica | 37.5 | 2 | 0 | 1 | Ratio of enablers to barriers is either 2:1 or 3:2 |
| Saint Vincent & Grenadines | 0.625 | 3 | 1 | 1 |  | Cote d'Ivoire | 37.5 | 3 | 1 | 1 |  |
| Serbia | 0.625 | 4 | 2 | 1 |  | Maldives | 37.5 | 3 | 1 | 1 |  |
| Sudan | 0.625 | 1 | 0 | 0 |  | New Zealand | 37.5 | 3 | 1 | 1 |  |
| Vietnam | 0.625 | 4 | 3 | 0 |  | Saint Vincent & Grenadines | 37.5 | 3 | 1 | 1 |  |
| Albania | 0.75 | 4 | 2 | 0 | 3 or four legal grounds present; enablers outweigh barriers by 2 | Albania | 50 | 4 | 2 | 0 | Four legal grounds present, enablers outweigh barriers by 2 |
| Benin | 0.75 | 4 | 1 | 1 |  | Benin | 50 | 4 | 1 | 1 |  |
| Burundi | 0.75 | 3 | 0 | 1 |  | Denmark | 50 | 4 | 2 | 0 |  |
| Denmark | 0.75 | 4 | 2 | 0 |  | Kyrgyzstan | 50 | 4 | 1 | 1 |  |
| Kyrgyzstan | 0.75 | 4 | 1 | 1 |  | Lao PDR | 50 | 4 | 2 | 0 |  |
| Lao PDR | 0.75 | 4 | 2 | 0 |  | Mali | 50 | 4 | 1 | 1 |  |
| Mali | 0.75 | 4 | 1 | 1 |  | Mauritius | 50 | 4 | 1 | 1 |  |
| Mauritius | 0.75 | 4 | 1 | 1 |  | Namibia | 50 | 4 | 1 | 1 |  |
| Namibia | 0.75 | 4 | 1 | 1 |  | Nepal | 50 | 4 | 1 | 1 |  |
| Nepal | 0.75 | 4 | 1 | 1 |  | Sierra Leone | 50 | 4 | 1 | 1 |  |
| Sierra Leone | 0.75 | 4 | 1 | 1 |  | Togo | 50 | 4 | 1 | 1 |  |
| Togo | 0.75 | 4 | 1 | 1 |  | Zambia | 50 | 4 | 1 | 1 |  |
| Zambia | 0.75 | 4 | 1 | 1 |  | Burundi | 56.25 | 3 | 0 | 1 | Ratio of enablers to barriers is 3:1 |
| Belarus | 0.875 | 4 | 1 | 0 | Four legal grounds present, 1 barrier | Belarus | 75 | 4 | 1 | 0 | Four legal grounds present, 1 barrier |
| Barbados | 0.875 | 4 | 1 | 0 |  | Barbados | 75 | 4 | 1 | 0 |  |
| Cambodia | 0.875 | 4 | 1 | 0 |  | Cambodia | 75 | 4 | 1 | 0 |  |
| Chad | 0.875 | 4 | 1 | 0 |  | Chad | 75 | 4 | 1 | 0 |  |
| Colombia | 0.875 | 4 | 0 | 1 |  | Colombia | 75 | 4 | 0 | 1 |  |
| Finland | 0.875 | 4 | 1 | 0 |  | Finland | 75 | 4 | 1 | 0 |  |
| Georgia | 0.875 | 4 | 1 | 0 |  | Georgia | 75 | 4 | 1 | 0 |  |
| Greece | 0.875 | 4 | 1 | 0 |  | Greece | 75 | 4 | 1 | 0 |  |
| Guyana | 0.875 | 4 | 1 | 0 |  | Guyana | 75 | 4 | 1 | 0 |  |
| Kazakhstan | 0.875 | 4 | 1 | 0 |  | Kazakhstan | 75 | 4 | 1 | 0 |  |
| Latvia | 0.875 | 4 | 1 | 0 |  | Latvia | 75 | 4 | 1 | 0 |  |
| Lithuania | 0.875 | 4 | 1 | 0 |  | Lithuania | 75 | 4 | 1 | 0 |  |
| Montenegro | 0.875 | 4 | 1 | 0 |  | Montenegro | 75 | 4 | 1 | 0 |  |
| Netherlands | 0.875 | 4 | 1 | 0 |  | Netherlands | 75 | 4 | 1 | 0 |  |
| South Africa | 0.875 | 4 | 1 | 0 |  | South Africa | 75 | 4 | 1 | 0 |  |
| Ukraine | 0.875 | 4 | 1 | 0 |  | Ukraine | 75 | 4 | 1 | 0 |  |
| Armenia | 1 | 4 | 0 | 0 | Four legal grounds present, no barriers | Armenia | 100 | 4 | 0 | 0 | Four legal grounds present, no barriers |
| Czechia | 1 | 4 | 0 | 0 |  | Czechia | 100 | 4 | 0 | 0 |  |
| DPRK | 1 | 4 | 0 | 0 |  | DPRK | 100 | 4 | 0 | 0 |  |
| Guinea Bissau | 1 | 4 | 0 | 0 |  | Guinea Bissau | 100 | 4 | 0 | 0 |  |
| Sweden | 1 | 4 | 0 | 0 |  | Sweden | 100 | 4 | 0 | 0 |  |
| Uruguay | 1 | 4 | 0 | 0 |  | Uruguay | 100 | 4 | 0 | 0 |  |

**
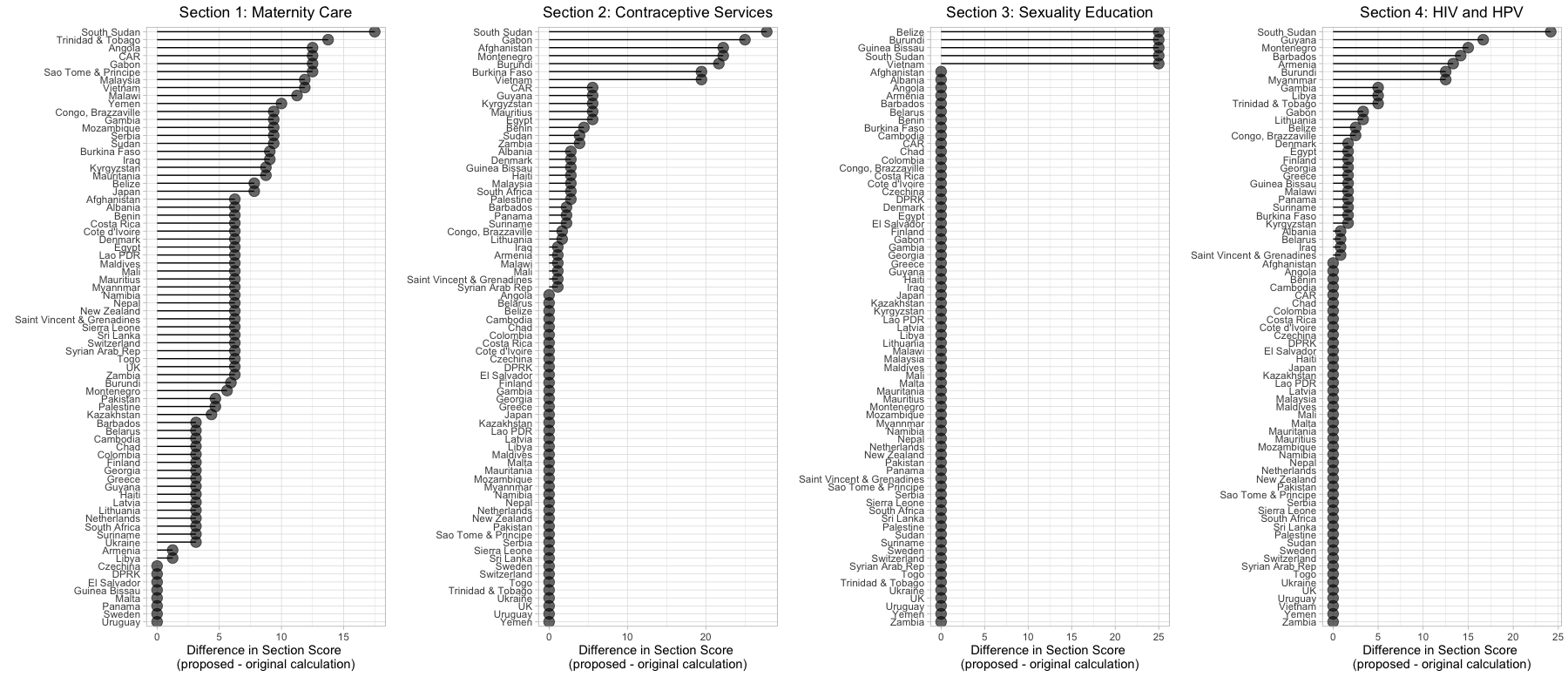
**

**Fig A: Differences in section scores between the current and proposed calculation of SDG Indicator 5.6.2 by country**

Note: Countries are ordered from largest to smallest difference in section score.

**
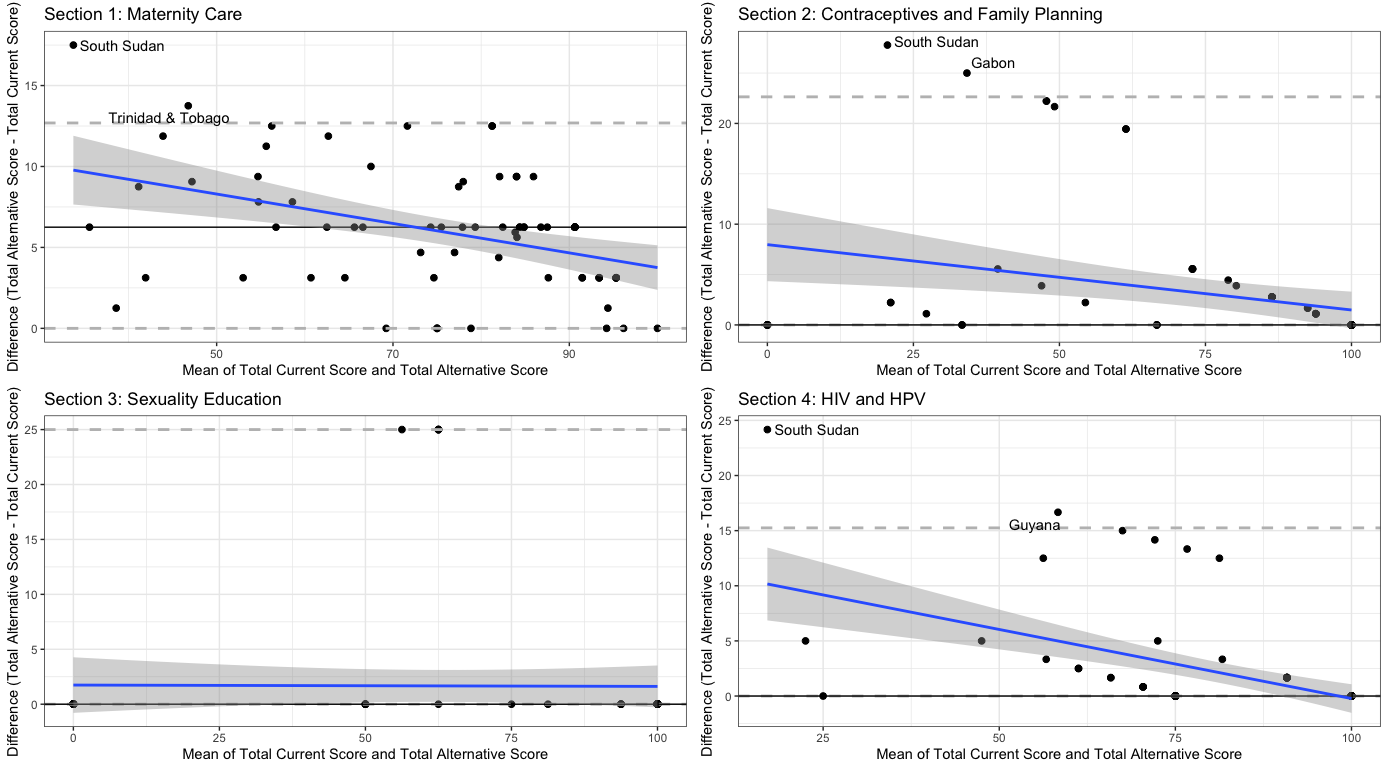
**

**Fig B: Bland-Altman plot showing the differences in section scores between the current and alternative calculation of SDG Indicator 5.6.2 by country by section**
